# Exploring the impact of mentoring functions on job satisfaction and organizational commitment of new staff nurses

**DOI:** 10.1186/1472-6963-10-240

**Published:** 2010-08-16

**Authors:** Rhay-Hung Weng, Ching-Yuan Huang, Wen-Chen Tsai, Li-Yu Chang, Syr-En Lin, Mei-Ying Lee

**Affiliations:** 1 Department of Hospital and Health Care Administration, Chia Nan University of Pharmacy and Science, Taiwan; 2 Department of International Business and Trade, Shu-Te University, Taiwan; 3 Department of Health Services Administration, China Medical University, Taiwan; 4 Department of Nursing, Jen-Ai Hospital, Taiwan; 5 Department of Nursing, Saint Paul's Hospital, Taiwan; 6 Department of Nursing, St. Martin De Porres Hospital, Taiwan

## Abstract

**Background:**

Although previous studies proved that the implementation of mentoring program is beneficial for enhancing the nursing skills and attitudes, few researchers devoted to exploring the impact of mentoring functions on job satisfaction and organizational commitment of new nurses. In this research we aimed at examining the effects of mentoring functions on the job satisfaction and organizational commitment of new nurses in Taiwan's hospitals.

**Methods:**

We employed self-administered questionnaires to collect research data and select new nurses from three regional hospitals as samples in Taiwan. In all, 306 nurse samples were obtained. We adopted a multiple regression analysis to test the impact of the mentoring functions.

**Results:**

Results revealed that career development and role modeling functions have positive effects on the job satisfaction and organizational commitment of new nurses; however, the psychosocial support function was incapable of providing adequate explanation for these work outcomes.

**Conclusion:**

It is suggested in this study that nurse managers should improve the career development and role modeling functions of mentoring in order to enhance the job satisfaction and organizational commitment of new nurses.

## Background

Nurse turnover and turnover intent have received considerable worldwide attention because of their influence on patient safety and health outcomes. The turnover intent among new nurses is often higher than that among senior nurses[1]. Nurse turnover in the first year of practice ranges between 35% and 60%[2]. With the high turnover incidence among new nurses, it is imperative that retention strategies be effective and that these strategies be examined closely. When new nurses perform their duties in hospitals, they often have little or no experience in clinical nursing but they are required to bear full responsibility of patient care. Owing to limited clinical skills, experience, and full responsibility of patient care, new nurses would often bear heavy work pressure. Work pressure and nurse attitudes toward jobs have significant impact on job satisfaction and organizational commitment among hospital nurses [3-5]. Researches from various countries have confirmed that job satisfaction and organizational commitment are statistically significant predictors of nurse absenteeism or turnover, or their intent to quit[1,6,7]. Thus, in order to reduce the nurses' intent to leave, nurse managers should urgently address the issue of improving the job satisfaction and organizational commitment of new nurses.

Proenca and Shewchuck[8] indicate that learning and career development opportunities are two important factors that would influence the retention of new nurses. Mentors play a vital role in providing these opportunities. Moreover, mentors can facilitate professional socialization of the new nurses in nursing; facilitate their transition into the workplace and social culture of the organization; and make them feel welcome in peer groups, with coworkers and the organization[9]. In addition, mentoring can promote the transfer of knowledge and values that support a hospital's mission. Therefore, a mentoring program is seen as a useful approach in improving the retention of new nurses[10,11].

The mentoring program is a formal relationship between a senior nurse and junior nurses of a hospital directed toward the advancement and support of the junior nurses[2,9]. It is a useful approach for new nurses as it provides them with effective and systematic support in the nursing practice, facilitates their professional development, and enhances the coordination of care within the unique context of general practice[12]. Tourigny and Pulich[13] argue that apart from the positive influence on medical care quality, mentoring programs play an important role in improving the work performance of the new nurses and establishing their attitudes toward the organizations they work for. Therefore, after the implementation of the new nurse mentoring program, mentors provide career development advice, psychosocial support, and role modeling functions to their mentees[9,10,12,14].

Psychosocial support functions include acceptance, counseling, and friendship. Friendship is provided by informal interactions at work, and by a willingness to discuss a variety of topics. Career development functions include sponsorship, protection, challenging assignments, exposure, and visibility. Exposure and visibility involve creating opportunities where important decision makers can observe and appreciate a person's competence, abilities, and special talents. Career development functions focus on the organization and the mentee's career, whereas psychosocial support functions affect the mentee at a more personal level and extend to other areas of life[15-17]. Besides, mentees have observed that mentors play a significant role in shaping their views on how they would act as mentors, thus highlighting the importance of role modeling. Role modeling is behaving and acting in a way that others can emulate; a role model displays appropriate attitudes, values, and behaviors to learn and follow[18-20]. In other words, role modeling functions focus on the fact that mentees would try to imitate the mentor's behavior because of their respect for and trust in the mentors.

Although it has been proven in previous studies that the implementation of a mentoring program is beneficial in terms of enhancing nursing skills and attitudes, few researchers have explored the impact of mentoring functions on the job satisfaction and organizational commitment of new nurses. Hence, the aim of this paper was to examine the effects of the different mentoring functions on the work outcomes through a survey of new nurses in Taiwan.

## Methods

### 1 Data Source and Analysis

We employed self-administered questionnaires to collect research data. For our subjects, we selected new nurses from three regional hospitals in Taiwan. In the course of the study, the participating hospitals facilitated formal meetings with head nurses, provided contact information on 308 eligible nurses who had been working in their hospital for two years or less, and gave their full support and coordination in the conduct of the research. We sent out 308 questionnaires over two months to survey the new nurses anonymously.

In all, 306 nurse subjects were obtained with an overall valid response rate of 99.35%. Because all the research data are self-reported and collected through the same questionnaire during the same period of time, a common method variance (CMV) may result in a systematic measurement error and may further bias the estimates of the true relationship between the theoretical constructs[21]. We have adopted some procedural techniques designed to address the CMV problem. These included protecting respondent anonymity, and reorganizing and trimming scale items. Besides, we also used Harman's one-factor test to check for the presence of CMV in the data. The Harman's one-factor test results were as follows: twenty factors had an eigenvalue greater than 1, and forty-four factors accounted for 97.85% of the variance. Thus, CMV was not considered a serious threat to this study[21]. After testing for CMV, reliability, and validity, we conducted a multiple regression analysis to test the impact of the mentoring functions.

### 2 Ethical consideration

The ethical issues of this study were reviewed and approved by the ethical committee of three sample hospitals. During the process of questionnaire collection, we also sent out informed consent form to 308 eligible nurses to be sure that they agreed to participate in this survey.

### 3 Measurement

#### 3.1 Research Variables

Questionnaire items were designed based on existing theoretical constructs and literature. Three mentoring experts and six nurse managers made some modifications and the contents of the questionnaires were validated by experts. The five-point Likert scale (1 = do not agree at all or not satisfied at all; 5 = extremely agree or extremely satisfied) was used as the measurement method. In addition, the construct validity of the questionnaire was tested by the confirmatory factor analysis.

3.1.1 Mentoring Function: Mentoring function is defined as the sum of the career development function, psychosocial support function, and role modeling function as perceived by nurses in the mentoring program. The measuring scales were based on the mentoring function scales proposed by Scandura[19], Raabe et al.[22], Sosik & Godshalk[20], and Eby et al[23]. Nine items were prepared, including three items in three dimensions: career development function, psychosocial support function, and role modeling function with a Cronbach's α value of 0.912. The mean value of the career development function, psychosocial support function, and role modeling function is 3.49 (SD = 0.64), 3.49 (SD = 0.73), and 3.90 (SD = 0.66), respectively.

3.1.2 Job satisfaction: After referencing literatures on job satisfaction[17,20,24,25], and evaluating the nurses' work responsibilities and experience, job satisfaction is defined as the nurses' overall state of satisfaction. Lankau & Scandura[24] point out that the Minnesota Satisfaction Questionnaire (MSQ) is a well-constructed scale for measuring work satisfaction in the mentoring program. Therefore, we modified the MSQ and created a total of five items with a Cronbach's α value of 0.865 and a mean of 3.59 (SD = 0.57).

3.1.3 Organizational commitment: Organizational commitment refers to the belief in and acceptance of organizational goals and values such that nurses are willing to make considerable efforts to achieve them, and are willing to remain with the organization[21,25-27]. The Organization Commitment Questionnaire (OCQ) developed by Mowday et al.[28] is accepted as the most widely used unidimensional measure of organizational commitment. It contains three dimensions: value commitment, effort commitment, and retention commitment[26]. Therefore, we developed a total of six items with a Cronbach's α value of 0.913 based on the OCQ and a mean of 3.40 (SD = 0.63).

#### 3.2 Control Variables

We used sample source, mentee's age[24,29], mentee's gender[24], mentee's education level[24], mentee's nursing experience[24,30], mentor's nursing ladder[20,30], mentoring period[24,29], and frequency of interactions with the mentor[24,29,30] as control variables in our regression models. Previous literatures have highlighted these variables and their significant influence on job satisfaction or organizational commitment. Frequency of interactions with the mentor was defined as the mentee's perceived degree of frequency rate of interaction with mentor per month. With respect to the measurement method, the five-point scale (1 = Seldom; 2 = Sometimes; 3 = Normally; 4 = Usually; 5 = Always) was used. Nursing experts involved in the study suggested that the factors "mentor had been trained for mentoring" and "mentor had the experience of mentoring" affect the work outcome of the new nurses in the mentoring program. Therefore, we included these two factors as control variables in the follow-up regression.

#### 3.3 Measure Validity and Reliability

All measures were subjected to a confirmatory factor analysis (CFA) using the AMOS 6.0 to test for unidimensionality, and convergent and discriminant validity[31]. Since job satisfaction does not include different dimensions, this study divided the five items of this construct into two composite indicators: the average value for the odd number items is named "job satisfaction 1," and the average value for the even number items is named "job satisfaction 2." After the CFA analysis, this model generated acceptable fit indices (χ^2^/df = 2.535; GFI = 0.969; AGFI = 0.931; RMR = 0.010; CFI = 0.988; NFI = 0.981; RFI = 0.967; IFI = 0.989; TLI = 0.980; RMSEA = 0.071). Table 1 show that the minimum value of the composite reliability of all the research constructs is 0.89 and the minimum average variance extracted (AVE) is 0.74. This indicates that every research construct possesses good internal consistency. In addition, Table 1 shows that the factor loadings of all the research construct items reach statistically significant levels. This indicates that the measurement model has good convergent validity[32]. The CFA results also showed that the square roots of all the AVE values of every research construct are higher than the pairwise correlation coefficients; the correlation coefficients between mentoring function and organizational commitment, between mentoring function and job satisfaction, and between organizational commitment and job satisfaction are 0.54, 0.63, and 0.75, respectively. This also shows that the measurement model of all the research constructs had good discriminate validity[32].

**Table 1:** Assessment of Convergent and Discriminant Validity (n = 306)

Construct and item	Factor loading	Error variance	Composite reliability	AVE
***Mentoring function***			0.89	0.74
Role modeling	0.84**	0.30		
Career development	0.96**	0.09		
Psychosocial support	0.78**	0.40		
***Organizational commitment***			0.94	0.85
Value commitment	0.94**	0.12		
Effort commitment	0.93**	0.13		
Retention commitment	0.89**	0.21		
***Job satisfaction***			0.90	0.83
Job satisfaction 1	0.89**	0.20		
Job satisfaction 2	0.92**	0.15		

Factor loadings are standardized. ** p < 0.01.

## Results

The mean age of the sample was 26.83 (SD = 3.87). Thirty-five percent of the sample had earned a Bachelor of Science degree or higher in nursing and seventy percent of the new nurses had nursing experience of more than one year. The mean period of the mentoring program was 3.97 (SD = 2.43), and the frequency rate of interaction between the mentors and mentees was 3.60 (SD = 0.86). In particular, most mentors earned the level III of the nursing ladder or higher (69.93%), had been trained for mentoring (62.09%), and had prior mentoring experience (83.66%) (Table 2). Besides, other summary statistics from the survey were shown in Table 3.

**Table 2:** Frequencies and Percentages on Selected Respondent Characteristics (n = 306)

Variable	Frequency	%	Variable	Frequency	%
Sample Source			Mentor's nursing ladder		
Hospital A	81	26.47	Level I	31	10.13
Hospital B	108	35.29	Level II	61	19.93
Hospital C	117	38.24	Level III	119	38.89
Sex			Level IV	83	27.12
Female	301	98.37	Level V	12	3.92
Male	5	1.63	Mentor had been trained for mentoring		
Education level			No	14	4.58
AA and lower	199	65.03	Yes	190	62.09
BS or higher	107	34.97	Unknown	102	33.33
Nursing experience			Mentor had the experience of mentoring		
< 3 mo	32	10.46	No	13	4.25
4-6 mo	31	10.13	Yes	256	83.66
7-12 mo	27	8.82	Unknown	37	12.09
1 to 2 yr	94	30.72			
2 to 3 yr	24	7.84			
> 3yr	98	32.03			

** p < .05

**Table 3:** Means, Standard Deviation and Correlation Matrix for Research Constructs

Variable	Means	SD	(A)	(B)	(C)	(D)	(E)
Role Modeling Function (A)	3.90	0.66	1				
Career Development Function (B)	3.49	0.64	0.64**	1			
Psychosocial Support Function (C)	3.49	0.73	0.59**	0.63**	1		
Job Satisfaction (D)	3.59	0.57	0.54**	0.54**	0.42**	1	
Organizational Commitment (E)	3.40	0.63	0.48**	0.49**	0.37**	0.70**	1
** p < .05							

During the survey period, we found that all the hospitals that were part of the study have an existing formal program that required mentors to give guidance and assistance to new staff nurses for at least two months. We found that the new staff obtained clinical guidance and mentor assistance for nearly four months, during which interaction with their mentors was frequent. Nursing managers usually assigned senior nurses--who had achieved the level III of the nursing ladder or higher--as mentors to guide the new staff nurses. Besides, mentors often not only had to be trained for mentoring but also usually had to have mentoring experience. Training program for mentoring in Taiwan is often designed for mentors. The types of this program include classes, seminar, workshop, or conference. The topics of all types of this program are related to the mentor's role and responsibilities, the mentor's communication skills, the mentor's clinical evaluation ability, mentor's case teaching ability, mentor's teaching evaluation and feedback, medical ethics and laws, or mentor's experience sharing. Therefore, there were some requirements related to the mentor's qualifications and these not only focused on clinical expertise but also placed importance on the mentoring abilities and experiences of senior nurses who wanted to be mentors.

Overall, the current mentoring program for new staff nurses actually works as intended. Respondents generally felt that this program is indeed capable of producing the effects of the career development, psychosocial support, and role modeling functions on them. Of these three functions, the level of the role modeling function as perceived by the mentees is the highest (mean = 3.90) as compared with the levels of the career development and psychosocial support functions, which are relatively lower (mean = 3.49). Thus, the mentors who served as senior nurses actually produced a role modeling effect on the new nurses; however, the mentees perceived relatively limited functions that concerned the psychological and development of new nurses (including career development and psychosocial support functions). We should focus on this finding and view it as a critical issue that requires our efforts to improve in the future.

Before conducting the multiple regression analysis, we adopted the Kolmogorov- Smirnov test to examine the residual normality of the regression models. The result of the Kolmogorov-Smirov test showed that the residual normality of each regression model was acceptable (p > 0.05). Besides, the residual analysis plot showed that our regression models did not violate the assumptions of linearity and homogeneity. We also assessed the multicollinearity of the models by examining the variance inflation factor (VIF) and found that all VIF values for independent variables were less than 7. This suggested that the multicollinearities of each regression model were not serious.

Table 4 shows the results of our multi-regression analysis for job satisfaction. The result of Model 1 indicates that three control variables--sample source, nursing experience, and frequency of interaction with the mentor--would significantly affect job satisfaction. After including the variables of the mentoring functions, only the career development function (β = 0.31) and the role modeling function (β = 0.30) were found to be significantly and positively related to job satisfaction; however, the coefficient of the psychosocial support function was not significant (Model 2). The results of the regression analysis for organizational commitment showed that four control variables--sample source, nursing experience, mentor had prior mentoring experience, and frequency of interactions with the mentor--would have a significant influence on organizational commitment (Model 1). After including the variables of the mentoring functions, we also found that the impact of the career development function (β = 0.28) and the role modeling function (β = 0.26) on organizational commitment is significantly positive, but the coefficient of the psychosocial support function is not significant (Model 2).

**Table 4:** Results of multiple regression analysis^a ^(n = 306)

Variable	Job Satisfaction				Organizational Commitment			
	
	Model 1		Model 2		Model 1		Model 2	
	β	Std. Error	β	Std. Error	β	Std. Error	β	Std. Error
**Sample Source**								
Hospital A^(R)^								
Hospital B	-0.22***	0.09	-0.17***	0.08	-0.27***	0.10	-0.23***	0.09
Hospital C	-0.08	0.09	-0.04	0.07	-0.11	0.10	-0.08	0.09
**Age**	0.13	0.01	0.09	0.01	0.20***	0.01	0.17***	0.01
**Gender**								
Female^(R)^								
Male	0.00	0.25	-0.03	0.22	-0.01	0.27	-0.03	0.25
**Education level**								
AA and lower^(R)^								
BS or higher	0.01	0.07	0.02	0.06	-0.05	0.08	-0.04	0.07
**Nursing experience**								
< 3 mo^(R)^								
4-6 mo	-0.12	0.14	-0.05	0.12	-0.17**	0.16	-0.12	0.14
7-12 mo	-0.12	0.15	-0.07	0.13	-0.16**	0.16	-0.12	0.14
1 to 2 yr	-0.19**	0.12	-0.09	0.10	-0.34***	0.13	-0.25***	0.12
2 to 3 yr	-0.14	0.15	-0.08	0.13	-0.22***	0.17	-0.17***	0.15
> 3yr	-0.31***	0.14	-0.11	0.12	-0.45***	0.15	-0.28***	0.14
**Mentor's nursing ladder**								
Level I^(R)^								
Level II	-0.03	0.13	0.02	0.11	-0.01	0.14	0.04	0.13
Level III	0.14	0.12	0.12	0.10	0.14	0.13	0.13	0.12
Level IV	0.08	0.12	0.07	0.11	0.06	0.13	0.06	0.12
Level V	-0.01	0.19	0.00	0.17	0.03	0.21	0.04	0.19
**Mentoring period (Mo)**	-0.06	0.01	-0.06	0.01	-0.03	0.01	-0.03	0.01
**Mentor had been trained for mentoring**								
No^(R)^								
Yes	-0.04	0.16	-0.04	0.14	-0.19	0.18	-0.19	0.16
Unknown	-0.11	0.17	-0.05	0.15	-0.22	0.19	-0.16	0.17
**Mentor had the experience of mentoring**								
No^(R)^								
Yes	0.11	0.17	-0.02	0.15	0.25**	0.19	0.14	0.17
Unknown	0.10	0.19	0.02	0.17	0.24**	0.21	0.18	0.19
**Frequency of interacting with the mentor**	0.23***	0.04	-0.02	0.04	0.21***	0.04	0.00	0.04
**Mentoring function**								
Role modeling			0.31***	0.06			0.26***	0.07
Career development			0.30***	0.06			0.28***	0.07
Psychosocial support			0.05	0.05			0.03	0.06

R^2^	0.15		0.39		0.18		0.36	
Adj.R^2^	0.09		0.34		0.12		0.30	
F Change	2.47***		36.81***		3.13***		25.55***	

^a ^Standardized regression coefficients are shown in the table

** p < .05; *** p <.01;^(R) ^reference group

## Discussion

Bahniuk[33] and Allen[34] have proven that the mentoring program enhances the job satisfaction of the mentees. During the mentoring process, mentors would often assign challenging and learning tasks to mentees in order to improve the mentees' knowledge and skills, provide career guidance, support the advancement of job position, help in resolving task-related problems, and further promote their overall growth. In this way, mentees improve their knowledge and skills and have a clear picture about their career development and position advancement [35-37]. The knowledge and experience exchange and learning opportunities in the mentorship were found to increase the mentees' sense of confidence toward their job, decrease their anxiety for the future, satisfy their career development needs and further create a high level of job satisfaction [7,19,38]. All the above mentioned benefits for mentees primarily stem from the career development function. Furthermore, McNeese-Smith & Nazarey[39] had used content analysis to analyze the interview data of 30 nurses. They found that apart from personal factors, the learning opportunities provided by the organization are critical factors that have a highly positive relationship with organizational commitment. In addition to providing a number of learning opportunities, mentoring programs also provide insight into the hospital systems and regulations; create room for performance feedbacks, discussions, and feedback friendship and offer the necessary clinical support. These career-related functions are vital for establishing a sense of organizational identity and organizational belonging. The organizational commitment of mentees is positively related to their sense of organizational identity and organizational belonging. Fang[40] proposed and tested a dual-process model of organizational commitment that connects organizational practices and specific job characteristics to the emotions and cognitions of employees. He found that an individual's perceptions of organizational support are key emotional and cognitive processes that mobilize commitment in the workplace. Overall, study results have proven a significant positive relationship between career development function and job satisfaction or organizational commitment of new staff nurses.

We also found a significant positive relationship between role modeling function and job satisfaction or organizational commitment of new nurses. As a result of the trust and respect for the mentors, the mentees can try to imitate the mentor's behavior in mentorship (role modeling) and can then upgrade their expertise and skills[13,41,42]. Besides, the mentees' perception of such mentoring relationships as trustful and respectful will be beneficial for the mentees in improving their workplace experience, perceptions, and future expectations. Therefore, if mentoring programs produced role modeling functions, they could effectively improve the expertise and skills of new nurses. In addition, it would facilitate the mentee's adaptation to nursing jobs and nursing environments. If new staff nurses adapt well to their nursing jobs and nursing environments, they will have higher job satisfaction and few complaints against the hospitals, invest more effort and initiative in their work, and further strengthen their commitment to the hospitals[9,11]. Besides, if mentees can perceive a high level of role modeling function in the mentorship, it indicates that mentors have more referent power for mentees. In such conditions, there are fewer conflicts between mentors and mentees when the former guide the latter. The decline in conflicts is positively related to the growing improvement in the relationship between the mentors and mentees and in the learning effects from the mentorship. In addition, the mentees' commitment to their organizations would further strengthen and improve the mentor-mentee relationship and increase the benefits of mentoring [10,43].

## Conclusion

This cross-sectional study examined the influence of career development, psychosocial support, and role modeling functions on the job satisfaction and organizational commitment of new staff nurses.

The results indicated that the development of mentoring programs for new staff nurses in Taiwan has reached a mature state and that the interactions between new nurses and their mentors are frequent, useful, and enduring. To serve as mentors, nurse managers assigned senior nurses who had been trained for mentoring, had previous mentoring experience, and had at least achieved the level III of the nursing ladder. In addition, mentoring programs for new nurses are indeed capable of generating the role modeling, psychosocial support, and career development functions. It is worth noting that mentors do exhibit a higher degree of nursing knowledge and technical capabilities; thus, the role modeling function appears to be significant in the mentoring process. However, it is likely that nursing mentors are not concerned about the psychosocial needs (for example, high praise from their mentors and the establishment of friendship in the workplace) and career anxieties or directions of new nurses. Therefore, the psychosocial support and career development functions, as perceived by new nurses, appear to be lower in Taiwan. Despite being perceived to be relatively lower by the new nurses, these two functions positively influence their job satisfaction and organizational commitment. If nurse managers want to improve the mentoring programs for new staff nurses, they should encourage mentors towards the direction of lending psychosocial support, providing directions on career development, giving opportunities for self-expression and promotion, and assigning challenging tasks that provide more learning opportunities for the new nurses. Effective mentoring will reinforce the job satisfaction of the new nurses and their commitment to the hospital.

In addition, the results of this study show that the role modeling function provided by mentors will positively influence the job satisfaction and organizational commitment of new staff nurses. If the nursing mentors can exhibit a high degree of nursing professionalism and attitude, win the mentees' confidence and respect, and be seen as benchmarks, it will facilitate the mentee's adaptation to their jobs and workplaces and will further enhance their job satisfaction and improve their organizational commitment. Therefore, while selecting senior nurses as mentors for new nurses, nurse managers should carefully consider their expertise and abilities. Besides expertise and abilities, nursing managers should reinforce the mentor's professional attitudes about nursing care and assist in improving their mentoring abilities in order to strengthen the role modeling functions of the mentoring programs.

### Limitations and future research suggestions

Since this study has a cross-sectional design, it has certain empirical limitations with regard to the verification of the relationship between research constructs. Therefore, it is suggested that future researchers use a study with a longitudinal design to further consider the impact of mentoring functions on job satisfaction and organizational commitment. In addition, we used Harman's one-factor test to assess the seriousness of common method biases. Although test results indicated that CMV was not considered a serious threat to this study, we suggest that if future researchers can overcome the data-collection and time-limitation problems, they can try to use the temporal, proximal, psychological, or methodological separation of measurement to attenuate the CMV problem.

Furthermore, this study only surveyed new staff nurses of Taiwan's hospitals; therefore, the findings cannot be generalized to other countries. Future researchers may collect samples from different countries and continue to test the assumptions of this research. Finally, future researchers can continue to investigate from different perspectives in order to explore the influential factors and the impact of these factors on the different dimensions of work outcomes (such as patient safety performance) and further strengthen the integrity of the theory of mentorship in nursing.

## Competing interests

The authors declare that they have no competing interests.

## Authors' contributions

WRH conceived of the study and made substantial contributions to study design, data acquisition, and data analysis and result interpretation. He also took responsibility for drafting and revising the manuscript. HJY performed the statistical analysis, assisted in interpreting the results and writing the manuscript. TWC was involved in designing the study, interpreting the analysis results and providing many important directions for literature review and discussion. CLY, LMY, and LSE took responsibility for data collection, participated in the data analysis and contributed to discussions. All authors read and approved the manuscript.

## Appendix.1 Construct Measurement Items

### Mentoring Function

Career development

The mentor gave me many important assignments that provided me with opportunities to learn nursing skills, for example, case analysis.

The mentor provided me with many suggestions about career development and highlighted the relevant concerns.

The mentor gave me a lot of information about position advancement opportunities.

Psychosocial support

I would discuss my private concerns and problems with my mentor.

I actually see my mentor as my "real" friend.

After work, I still kept in touch with my mentor.

Role modeling function

I tried to adapt my mentor's behavior.

I really respect and admire my mentor's nursing expertise and skills.

I really respect and admire my mentor for her mentoring expertise and skills.

### Job Satisfaction

With regard to the promotion opportunities in this job, I feel.......

With regard to the sense of achievement while finishing my jobs, I feel.......

With regard to the performance feedbacks, I feel.......

With regard to whether my salary and welfare are commensurate with the workload, I feel.......

With regard to the interactions with my coworkers, I feel.......

### Organizational Commitment

Effort commitment

I am willing to put in extra efforts for my hospital.

I am willing to make an all-out effort to keep up with the hospital's development.

Value commitment

I feel a sense of pride in doing my job at this hospital.

My current work environment is the ideal one, which I always hoped to work in.

Retention commitment

Even though there are better opportunities outside, I will not leave this hospital.

I have strong commitment to continue providing my services at this hospital until retirement.

## Pre-publication history

The pre-publication history for this paper can be accessed here:

http://www.biomedcentral.com/1472-6963/10/240/prepub
